# A Transcription Factor Contributes to Pathogenesis and Virulence in *Streptococcus pneumoniae*

**DOI:** 10.1371/journal.pone.0070862

**Published:** 2013-08-13

**Authors:** Layla K. Mahdi, Esmaeil Ebrahimie, David L. Adelson, James C. Paton, Abiodun D. Ogunniyi

**Affiliations:** 1 Research Centre for Infectious Diseases, School of Molecular and Biomedical Science, The University of Adelaide, Adelaide, South Australia, Australia; 2 Centre for Bioinformatics and Computational Genetics, School of Molecular and Biomedical Science, The University of Adelaide, Adelaide, South Australia, Australia; University of Helsinki, Finland

## Abstract

To date, the role of transcription factors (TFs) in the progression of disease for many pathogens is yet to be studied in detail. This is probably due to transient, and generally low expression levels of TFs, which are the central components controlling the expression of many genes during the course of infection. However, a small change in the expression or specificity of a TF can radically alter gene expression. In this study, we combined a number of quality-based selection strategies including structural prediction of modulated genes, gene ontology and network analysis, to predict the regulatory mechanisms underlying pathogenesis of *Streptococcus pneumoniae* (the pneumococcus). We have identified two TFs (SP_0676 and SP_0927 [SmrC]) that might control tissue-specific gene expression during pneumococcal translocation from the nasopharynx to lungs, to blood and then to brain of mice. Targeted mutagenesis and mouse models of infection confirmed the role of SP_0927 in pathogenesis and virulence, and suggests that SP_0676 might be essential to pneumococcal viability. These findings provide fundamental new insights into virulence gene expression and regulation during pathogenesis.

## Introduction

Bacterial pathogens use different strategies and elicit a variety of virulence factors during infection of the host to establish disease. For instance, enteric pathogens such as *Escherichia coli*, *Salmonella, Shigella and Vibrio cholerae* employ a number of secretion systems and effector molecules to subvert their target host cells [1], [2]. In addition, during infection, pathogenic bacteria activate a number of transcription factors (TFs) which control the regulatory cascades that govern their physiological adaptation, pathogenesis and virulence [3]–[5]. For example, in *V. cholerae*, studies have shown that regulation of virulence gene expression is governed by ToxR and ToxT [2], [6]–[8]. However, the TFs, regulatory mechanisms, molecular networks and precise events that control the translocation of other important pathogenic bacteria such as *Streptococcus pneumoniae* (the pneumococcus) from the initial site of infection into deeper host tissues, are yet to be fully elucidated [9], [10]. Nevertheless, the pneumococcus continues to be responsible for high global morbidity and mortality resulting from pneumonia, bacteremia, meningitis and otitis media [11], largely due to our incomplete understanding of the biology of pneumococcal disease [12].

To address these deficiencies, we initially carried out systematic microarray comparisons of gene expression kinetics of two pneumococcal strains in the nasopharynx, lungs, blood and brain of mice. These analyses yielded a number of niche-specific, up-regulated genes that contribute to pathogenesis, some of which were shown to encode good vaccine candidates [13], [14]. Surprisingly, our investigations and similar *in vivo* transcriptomic analyses by others [13]–[15] did not identify any significantly up-regulated TFs, despite their prominent role in bacterial pathogenesis [3]. We reasoned that this is probably due to low, and generally transient, expression of TFs, although a small change in the expression or specificity of a TF can radically alter gene expression. Therefore, in this study, we utilized our existing transcriptomic data to comprehensively analyze TFs controlling the progression of pneumococci from the nasopharynx to deeper host tissues by comparing the ratio of expression of these genes between distinct host niches during pathogenesis.

## Materials and Methods

### Ethics Statement

Outbred 5- to 6-week-old female CD1 (Swiss) mice were used in all experiments. The Animal Ethics Committee of The University of Adelaide approved all animal experiments (Project Number: S-2010–001). The study was conducted in compliance with the Australian Code of Practice for the Care and Use of Animals for Scientific Purposes (7th Edition 2004) and the South Australian Animal Welfare Act 1985.

### Bacterial strains and growth conditions

The pneumococcal strains used in this study were clinical blood isolates WCH43 (serotype 4; Sequence Type 205) and WCH16 (serotype 6A; Sequence Type 4966). Previous mouse intranasal challenge experiments in our laboratory with both strains indicated that WCH43 is more virulent than WCH16. Nevertheless, both strains have a propensity to translocate to the brain of infected mice. Furthermore, WCH43 infection of mice demonstrates the “classical” disease progression from the nasopharynx to the lungs and dissemination to blood and then to the brain [16], [17]. However, WCH16 seems to progress directly to the brain with minimal lung and blood involvement, suggesting that the preferred route for WCH16 pathogenesis is by direct translocation into the brain via the nasopharyngeal epithelium. Serotype-specific capsule production was confirmed by Quellung reaction, as described previously [18]. Opaque-phase variants of the strains, selected on Todd-Hewitt broth supplemented with 1% yeast extract (THY)-catalase plates [19], were used in all animal experiments. Before infection, the bacteria were grown statically at 37°C in serum broth (SB) to *A*_600_ of 0.16 (equivalent to approx. 5×10^7^ CFU/ml).

### Analysis of *in vivo* microarray data of pneumococcal movement between different host tissues

For this analysis, we utilized microarray data of *in vivo*-derived RNA samples obtained from our previous studies [13], [14]. Two color microarray analysis was carried out where the relative expression of each gene in one niche was calculated in comparison to expression in the previous niche when bacteria moves from nasopharynx→lungs→blood→brain. The goal of this analysis was to unravel pneumococcal gene expression kinetics in the nasopharynx, lungs, blood and brain of mice. The following formula was used to calculate the relative expression of bacterial genes in each niche versus the previous niche: Log_2_ [(Rmean – morphR)/(Gmean – morphG)], where Rmeam and Gmean were mean of Red and Green intensities, respectively (foreground intensities) and morphG and morphR were Green and Red background intensities. FlexArray package (McGill University, Canada) was used to analyze microarray data.

In the context of gene selection, in addition to taking into account the amount of gene expression (quantity of expression, selected based on the relative fold change ≥2, and *P*-value of one-sample *t*-test ≤0.05), different strategies were employed to select genes based on quality of selection. The quality based gene selection strategies were: stability of gene expression, *in silico* structural analysis of overexpressed genes at the protein level and GO classification (using our recently developed comparative GO web application [20].

In the stability of gene selection strategy, we searched for the genes showing the relative stability of up-regulation between different niches. In other words, one criterion in gene selection considered the pneumococcal genes that were continuously up-regulated during transition from one niche to another (or at least maintained the same level of expression). Another quality-based gene selection strategy was protein structural prediction of up-regulated genes. This was carried out using CLC Main Workbench package (CLC bio company, Finland), ExPASY (http://expasy.org/), pfam (http://pfam.sanger.ac.uk), KEGG (http://www.genome.jp/kegg/), and Conserved Domains and Protein Classification database (http://www.ncbi.nlm.nih.gov/Structure/cdd/cdd.shtml). Regarding the lack of comprehensive study on TFs, particular attention was paid to finding up-regulated genes with helix-turn-helix/helix-loop-helix DNA binding and Zinc finger structures, since these structures are the common universal protein structure of TFs in all organisms. For gene network analysis, up-regulated genes during progression from the nasopharynx to lungs, blood and brain were used as input for making the networks. A database was built using Pathway Studio 9 software (Elsevier, USA), which contains different gene interaction information obtained from correlation expression analysis and literature mining.

### Functional catalogue of pneumococcal pathogenesis through classification of bacterial modulated genes into Gene Ontology (GO) groups in different host tissues

A comprehensive view of bacterial functional genomics can be obtained by categorizing up-regulated/down-regulated genes into a limited number of annotated GO groups. GO classification has been well developed for eukaryotes; however, it has not been extensively applied in understanding functional genomics of bacteria. Here, we assigned GO groups to up-regulated and down-regulated pneumococcal genes in both WCH16 and WCH43 using our recently developed comparative GO web application [20]. Specific attention was paid to GO classes involved in regulatory mechanisms such as sequence-specific DNA binding transcription factors, DNA binding, and two-component response regulator activity. We used this classification for increasing the quality of gene selection. More importantly, GO classification increased our knowledge about bacterial functional genome arrangement and shift during infection of different host tissues. GO categories were classified as: biological process, cellular component, and molecular function.

### The effect of SP_0927 mutation on whole transcriptomics and functional organization of pneumococcal genome

We were surprised that while the ratio in bacterial counts of ΔSP_0927 mutant in the blood versus lungs was not significantly different from that of the wild-type (WCH43), the mutant showed reduced virulence. Therefore, we investigated transcriptional changes that could be responsible for this phenomenon by microarray comparisons of RNA harvested from wild-type and ΔSP_0927 grown in SB to *A*_600_ = 0.16. Microarray experiments were performed on whole genome *S. pneumoniae* PCR arrays obtained from the Bacterial Microarray Group at St George's Hospital Medical School, London (http://bugs.sgul.ac.uk/). The array was designed using TIGR4 base strain annotation [21] and extra target genes from strain R6 [22]. The array design is available in BµG@Sbase (Accession No. A-BUGS-14; http://bugs.sgul.ac.uk/A-BUGS-14) and also ArrayExpress (Accession No. A-BUGS-14). Microarray probes were generated using the 3DNA Array 900 MPX labeling kit (Genisphere) following the manufacturer's guidelines. The fluorescently labeled cDNAs for the pair-wise comparison were then combined and hybridized to the surface of the microarray, essentially as described previously [23]. Microarray analysis was performed on a total of 3 independent hybridizations from three separate assays (including one dye reversal), essentially as described previously [13], [14], [23], [24]. The top 50 differentially expressed genes with *p*<0.05; (one sample *t*-test) using log_2_ (647/546) ratios from each hybridization were then subjected to functional catalogue classification using our new web application [20] to unravel the impact of mutating SP_0927 on pneumococcal functional genomics.

### Relative Quantitation real-time RT-PCR

For a subset of selected pneumococcal genes that were significantly differentially expressed between the nasopharynx, lungs, blood and brain (and in the *in vitro* comparison of WCH43 with its isogenic ΔSP_0927 mutant) by microarray analysis, relative gene expression were validated using a one-step Superscript III Platinum® qRT-PCR kit (Invitrogen) in a LightCycler®480 II (Roche) as described previously [13]. The relative gene expression was analyzed using the 2^−ΔΔCT^ method [25]. The reference gene was 16S rRNA. The primer pairs used for gene expression analysis are listed in Table S6. All data were obtained from three biological replicates.

### Construction of mutants and assessment of bacterial growth *in vitro*

*S. pneumoniae* derivatives with marked mutations in genes of interest were constructed in WCH43 (serotype 4). Mutants were constructed by overlap extension PCR as described previously [26] and validated by PCR and sequencing to be in-frame deletion mutation replacements. All PCR procedures were performed with the Phusion High Fidelity Kit (FINNZYMES). The primer pairs used for construction and validation of the mutants are listed in Table S6. In order to evaluate the growth rate of the mutants in comparison to the wild-type, bacterial strains were grown in SB and *A*_600_ monitored overnight on a Spectramax M2 spectrophotometer (Millenium Science). For *in vitro* competition experiments, mutant and wild-type bacteria were grown to *A*_600_ in SB and then mixed at an input ratio of 1∶1 in SB. At 1.5 and 3 h post incubation, an aliquot of each sample was serially diluted in SB and plated on blood agar and blood agar with a selective antibiotic to determine the ratio of mutant to wild-type bacteria. Each competition experiment was repeated at least twice. Competitive indices were calculated as the ratio (± SEM) of mutant to wild-type bacteria recovered at each time point adjusted by the input ratio.

### Pathogenesis and virulence assessment of mutants

For pathogenesis experiments, *S. pneumoniae* derivatives with mutations in genes of interest and the isogenic wild-type strain were grown separately in SB to *A*_600_ = 0.16 (approx. 5×10^7^ CFU/ml). For pathogenesis experiments, 8–10 mice were anesthetized by intraperitoneal injection of pentobarbital sodium (Nembutal; Rhone-Merieux) at a dose of 66 mg per g of body weight and separately challenged i.n. with 50 µl suspension containing approx. 2.5×10^6^ CFU of either wild-type or the isogenic mutant (ΔSP_0746 and ΔSP_0927). At 48 h post-challenge, mice from each separate infection experiment were sacrificed, bacteria were enumerated from the nasopharynx, lungs blood and brain, as described previously [13], [14] The experiment was carried out at least twice for each strain. Ratios of bacteria counts between niches were determined and verified to follow log-normal distribution by the log-normality test. Differences in ratios between wild-type and mutants were compared using unpaired two-sample *t*-test (one-tailed). To assess the virulence potential of mutants, groups of 12 anesthetized mice were challenged i.n. with either mutant or wild-type bacteria, as described previously [13], [27] Each mouse received 50 µl of bacterial suspension containing approximately 1×10^7^ CFU in SB. The challenge dose was confirmed retrospectively by serial dilution and plating of the inocula on blood agar. The survival of mice was monitored four times daily for the first 5 days, twice daily for the next 5 days, and then daily until 14 days after challenge. Differences in median survival times for mice between groups were analyzed by the Mann-Whitney *U*-test (one-tailed).

### Data Access

The data reported in this paper are archived at the following databases: BµG@Sbase (http://bugs.sgul.ac.uk/E-BUGS-130 and http://bugs.sgul.ac.uk/E-BUGS-133, and also ArrayExpress (accession number E-BUGS-130, and E-BUGS-133.

## Results and Discussion

In this investigation, we have subjected our existing transcriptomic data to bioinformatics and gene network prediction to identify other genes (particularly TFs) critical to invasive pneumococcal disease that have previously been missed by existing conventional gene identification strategies.

### Bioinformatic prediction of two TFs that potentially contribute to pathogenesis and virulence of *S. pneumoniae*

To characterize the tissue-specific pneumococcal TFs and genes under their regulation, we used a variety of statistical and bioinformatic techniques including correlation of expression, gene network reconstruction, microarray and qPCR analysis. Bioinformatic gene selection criteria also included stability of gene expression, *in silico* structural analysis of over-expressed genes for DNA-binding motifs (such as helix-turn-helix (HTH) motifs, Zinc finger motifs) and literature mining. In this manner, we found a complex activated network of genes governing pneumococcal virulence machinery commonly up-regulated during infection by clinical pneumococcal strains WCH43 (serotype 4) and WCH16 (serotype 6A), particularly between lungs and the nasopharynx, and between brain and blood. However, fewer genes were found to be regulated during pneumococcal progression from the lungs to blood (Table S1), in agreement with our previous observations [14].

**Figure 1:**
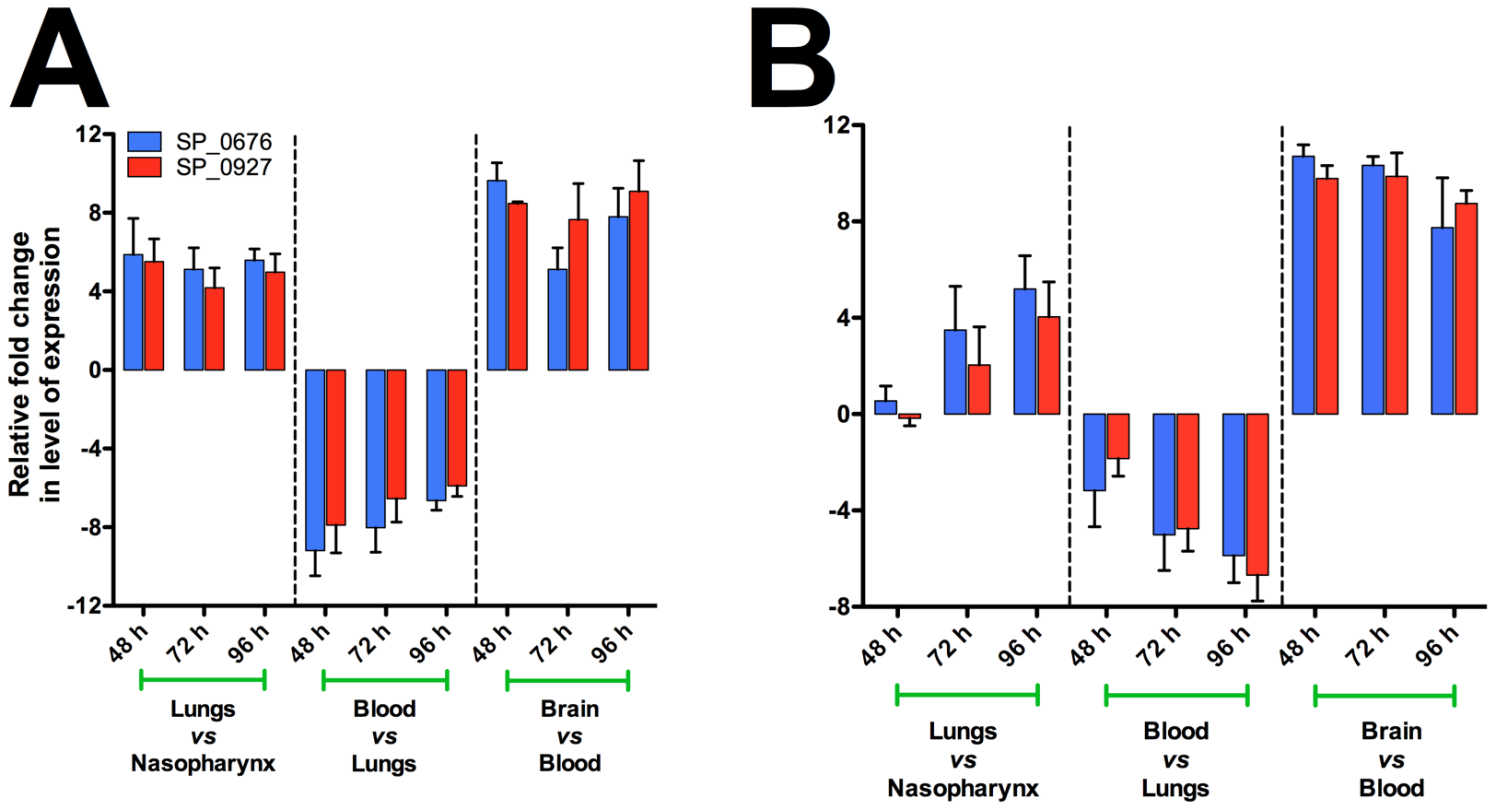
Expression and of SP_0676 and SP_0927 in *S. pneumoniae* WCH16 (A) and WCH43 (B), showing up-regulation and high correlation of both TFs in all niches except the blood.

**Figure 2:**
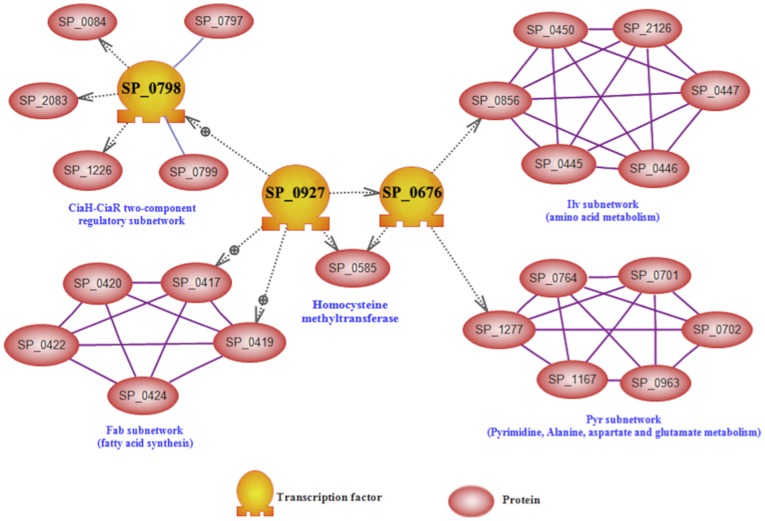
Bioinformatic prediction model for two TFs that potentially contribute to pathogenesis and virulence of *S. pneumoniae.* Gene regulatory network analysis of SP_0676 and SP_0927 in the brain versus blood. The interaction network consists of *ilv*, *pyr*, *fab* and *cia* subnetworks. Dashed arrows represent positive or negative regulation; dashed arrows with a ⊕ sign represents positive regulation.

Protein structural prediction of up-regulated genes and gene ontology (GO) classification using our recently developed comparative GO web application [20] resulted in the identification of two possible TFs influencing virulence, SP_0676 and SP_0927. These two TFs belong to the LysR-type transcriptional regulator family, which are highly conserved and ubiquitous among bacteria [28]. Bioinformatic analyses revealed that SP_0676 is a MtaR-type repressor, while SP_0927 (annotated as SmrC by other workers [29]–[31] is a CmbR-type repressor (or activator) in Streptococcaceae. Both possess HTH- DNA binding motifs, they were up-regulated in all niches except the blood in both strains, and their expression levels showed a high correlation with each other [Pearson correlation test; *P* = 0.001] (Figure 1). The two TFs also exhibit 32.6% amino acid identity. Bioinformatic prediction of the activated gene regulation network between lungs and the nasopharynx, and between brain and blood allows us to propose a model for a central role for these two TFs (Figure 2). The activated network can be divided into sub-networks such as *ilv* net (containing *ilvABCDEN*), *pyr* net (containing *pyrBDEF*), *fab* net (*fabDFHKZ*), and *cia* net (*ciaRH*, and *pepN*) (Figure 2; Table S1). We then conducted RT-PCR on *S. pneumoniae* mRNA of pneumococci from the blood and brain, to validate regulation of selected genes shown in our network prediction, which yielded consistent results with the bioinformatic prediction (not shown).

Other workers have characterized global regulation of gene expression in *S. pneumoniae* by many TFs including CcpA [32], CodY [33], Mga [34], [35], PsaR [36], [37], RitR [38], and two-component systems [39] under *in vitro* conditions. However, to our knowledge, niche-specific TF-modulation of virulence gene expression during pneumococcal movement from the nose to lungs, blood and brain directly attributable to these two TFs (SP_0676 and SP_0927), is yet to be reported in the literature. We then attempted to assess the contribution of both TFs to pathogenesis and virulence by targeted deletion-replacement mutagenesis. While a mutation in SP_0927 was achieved, extensive efforts to construct a mutant of SP_0676 using various pneumococcal transformation techniques and in different strains were unsuccessful, suggesting that a mutation in this gene could be lethal to the cell. This is in agreement with another recent study indicating that SP_0676 is essential [10].

**Figure 3:**
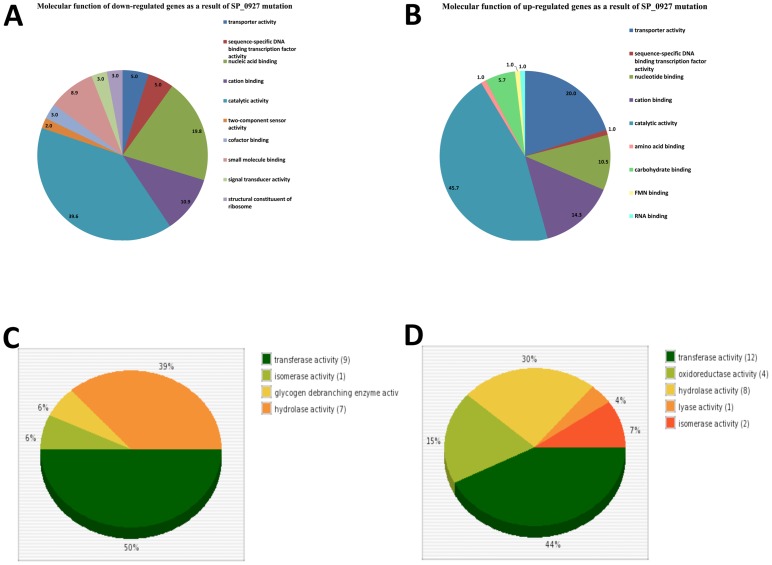
Molecular functional categories of genes (A), down-regulated, and (B), up-regulated in ΔSP_0927 mutant. (C and D), Comparison of the sub-categories of down-regulated (C) and up-regulated (D) genes due to SP_0927 mutation under “catalytic activity” molecular function group.

We then compared gene expression patterns of *in vitro*-grown WCH43 with that of its isogenic ΔSP_0927 mutant by microarray analysis. It was of interest that GO analysis of the differential gene expression patterns using our recently developed comparative GO web application [20] revealed that two-component sensor activity and DNA binding functional groups were down-regulated in SP_0927 mutant, while genes involved in carbon utilization and transporter activity were up-regulated (Figure 3, A and B; Table S2). The “catalytic activity” molecular function was predominant in both the up- and down-regulated gene functional categories. A sub-division of this category showed genes with common, as well as unique enzymatic activities (Figure 3, C and D).

### Functional catalogues of pneumococcal pathogenesis in different host tissues

**Figure 4:**
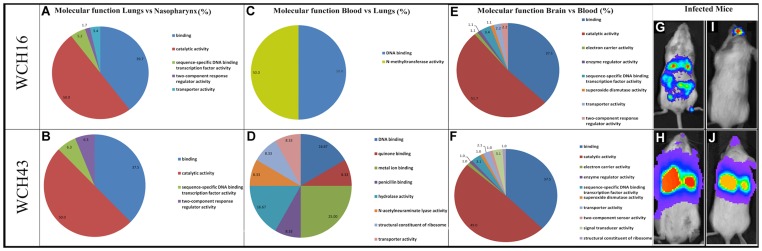
Functional genomics catalogues of *S. pneumoniae* WCH16 and WCH43 pathogenesis in different host tissues. (A and B), Lungs versus Nasopharynx; (C and D), Blood versus Lungs; (E and F), Brain versus Blood. (G–J): Bioluminescent imaging of WCH16- or WCH43- infected mice at 72 h post-challenge, showing bacteria in the nasopharynx, lungs, blood and brain. G and H; ventral views, I and J; dorsal views of infected mice.

As part of our investigation, we were interested in obtaining a more comprehensive picture of the overall functional relationships between the two identified TFs and other differentially regulated genes in various niches during pathogenesis of disease at 48, 72 and 96 h post-infection. Our assessment was facilitated by the comparative GO web application that we recently designed [20]. By analyzing the GO of up-regulated genes of WCH16 and WCH43 in different tissues at various times after infection, we found the highest increase of different functional groups in the brain versus blood (Table S1). This included genes encoding antioxidant enzymes (such as SP_0313, SP_0766 and SP_0784) and a large number of brain-specific TFs such as MalR, DNA-binding response regulator, arginine repressor, SP_0676 and SP_0927 (Table S3). We found that the acetyl-CoA carboxylase complex functional group (cellular component) predominated in both brain and blood, suggesting a role for fatty acid biosynthesis in pneumococcal pathogenesis. Interestingly, sequence-specific DNA binding TF activity was an over-represented GO in both lungs and brain (Figure 4, A–F), suggesting a key role for TFs in pneumococcal functional genomics. Functional classification of genes expressed by WCH16 and WCH43 during pathogenesis differed mainly in blood: more GO groups were activated in WCH43 (Figure 4, C and D), suggesting that these activated GO groups may be important in maintaining fulminant bacteraemia by this strain. We also found a major difference in gene expression between both strains in blood; whereas only 3 genes were up-regulated in the blood by WCH16 (relative to lungs), 16 genes were up-regulated in the blood relative to lungs in WCH43 (Table S4). These marked differences in gene expression patterns are consistent with the observed differences in the pathogenicity characteristics of the two strains [13], [16], [17] and also correlates with bioluminescence patterns of the two strains in mice (Figure 4, G–J). Together, these analyses have increased our knowledge of pneumococcal gene regulatory mechanisms and changes in gene expression patterns during pathogenesis. Moreover, GO categories of up-regulated genes of the two strains provide a comparative view of different functional genomics organizations of pneumococcal strains during pathogenesis (Figure 4, A–F).

### Identification of up-regulated pneumococcal genes critical to pathogenesis and virulence

**Figure 5:**
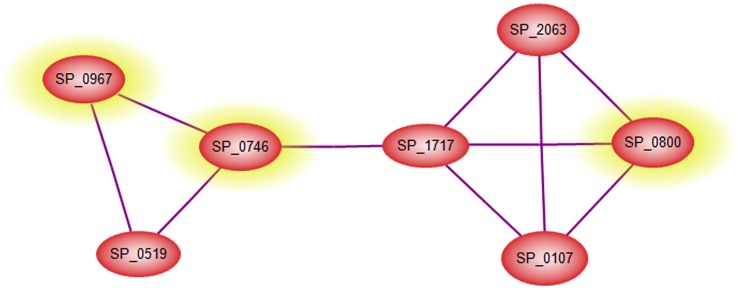
Interaction network data for connectivity of SP_0746 to SP_0800 and SP_0967 (highlighted in yellow).

Our search for genes that might be important to the disease process revealed 4 genes, SP_2089, SP_0569, SP_0967 and SP_0800, which showed high level of expression in both blood and brain. Two of these 4 genes are part of a highly connected network (Figure 5) suggesting that they could be good targets for blocking pneumococcal virulence machinery from the nasopharynx to the brain. Network analysis of these 4 genes revealed another gene, SP_0746 (“ClpP”) that was highly connected with these genes, suggesting that this gene might be involved (directly or indirectly) in the modulation of expression of the 4 genes. This led us to select it for further analysis.

**Figure 6:**
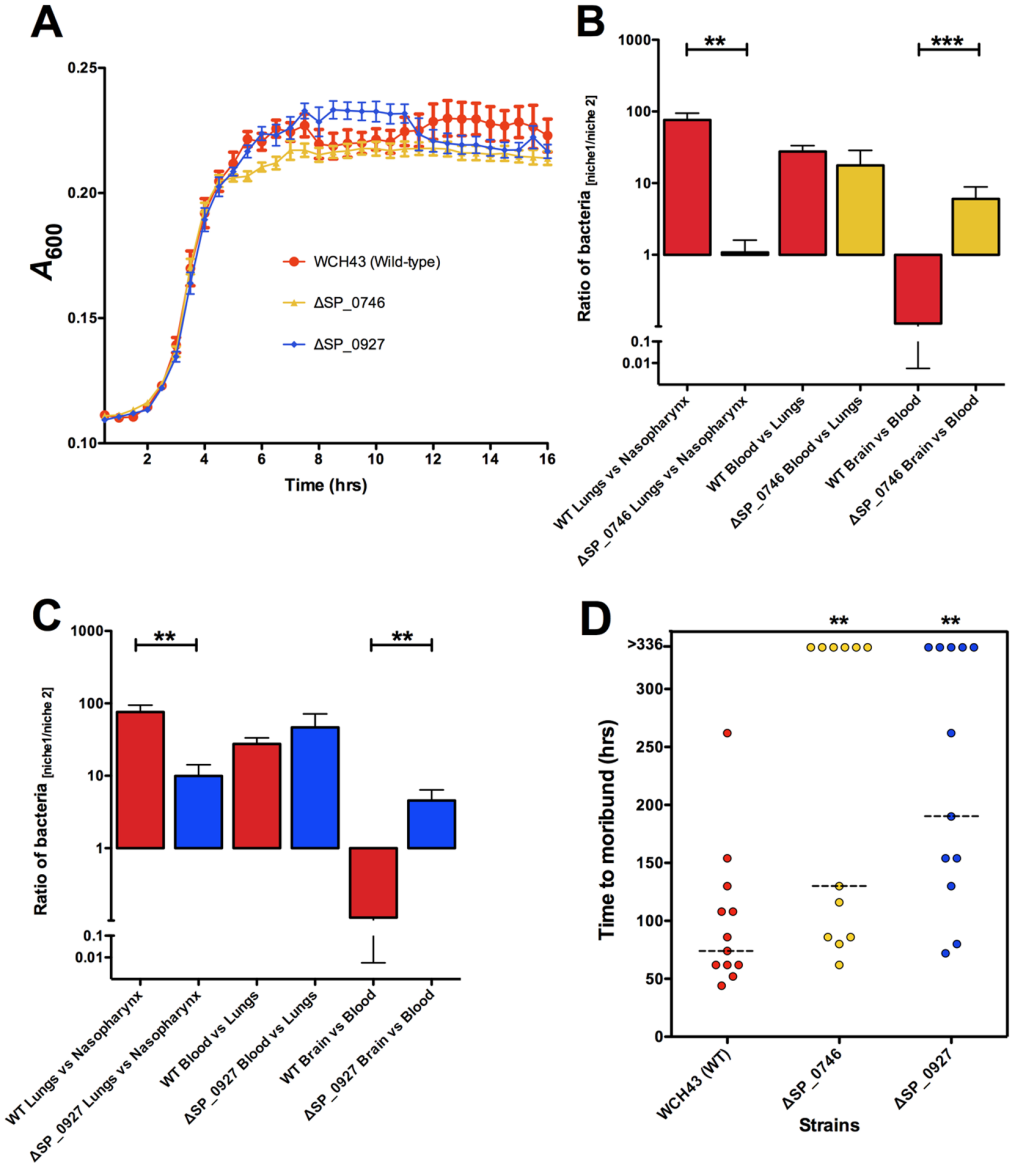
Characterisation of mutants of *S. pneumoniae* WCH43. (A), *In vitro* growth curves of mutants and wild-type. Data are mean ± SEM of 7 replicates for each time point for each strain. (B and C), Comparison of pathogenesis progress of mutants relative to wild-type; (B), ΔSP_0746; (C), ΔSP_0927. Data are mean ± SEM of pathogenesis ratios for 16–24 mice for the mutants, and 40 mice for wild-type. Data were analyzed by unpaired *t*-test, one tailed (**, *P*<0.01; ***, *P*<0.001). (D). Survival times for mice after intranasal (1×10^7^ CFU) challenge with mutants and wild-type. Each datum point represents one mouse. Broken lines denote median survival time for each strain. Mice that survived beyond 14 days are indicated at the top of the figure as >336. ** *P*<0.01; Mann-Whitney *U*-test, one-tailed. NS =  Not significant.

We then evaluated the contribution of selected genes preferentially up-regulated in various tissues to pathogenesis and virulence by targeted mutagenesis. Deletion replacement mutation of these genes (SP_0746, and SP_0927) did not adversely affect their growth in serum broth, either separately or in competition with the isogenic WCH43 wild-type strain (Figure 6A, Table S5). Consistent with network analyses, ratio of pathogenesis data confirmed that SP_0746 and SP_0927 contribute significantly to lung and brain infection (Figure 6, B and C). In support of these findings, intranasal challenge of mice showed that ΔSP_0746 and ΔSP_0927 mutants were significantly attenuated for virulence relative to wild-type (Figure 6D; *p*<0.01 in both cases). These data are consistent with attenuation of SP_0746 (ClpP) in previous findings, albeit in a different virulent (serotype 2 [D39]) genetic background [40]–[42]. Other workers have also demonstrated reduced virulence of a SP_0927 (“SmrC”) mutant generated by signature-tagged mutagenesis in pneumonia and bacteremic competition models in serotype 3 [29] and serotype 4 [30] pneumococci. The delay in the onset of mortality provides a window for host recovery and survival as well as adequate time for effective antibiotic therapy, as was indeed the case in protection and antibiotic treatment experiments with SP_0746 [43]. The reduced virulence of the ΔSP_0927 mutant correlates with the down-regulation of genes involved in virulence in WCH43, as found in our microarray analysis (Figure 3A). These findings reinforce the importance of TFs and the potential of network-based gene prediction in control of infectious diseases.

### Conclusion

Our comprehensive statistical and bioinformatic prediction of pneumococcal TFs and genes under their regulation revealed for the first time, a potential central gene network governing pneumococcal pathogenesis and virulence machinery. We also note that genes in the *pyr* net and *cia* net found in our predictive network analysis were also contemporaneously identified using Tn-seq, a high throughput screening strategy [10]. This not only validates our strategy, but also represents a convergence of novel strategies for a detailed understanding of bacterial pathogenesis. We suggest that calculating the ratio of bacterial gene expression in each host niche relative to the previous niche, in combination with selection based on absolute gene expression level, might provide an additional level of selection for genes critical to pathogenesis. We also suggest that the use of quality-based metrics such as GO classification, and network analysis in conjunction with quantity-based gene selection criteria is likely to be more robust for elucidating potential vaccine and therapeutic targets.

## Supporting Information

Table S1 **Highly up-regulated genes in** ***S. pneumoniae*** **WCH16 and WCH43 during pathogenesis.**
(DOCX)

Table S2 **List of top 50 significantly differentially-expressed genes as a result of SP_0927 mutation in** ***S. pneumoniae*** **WCH43 by microarray analysis.**
(DOCX)

Table S3 **List of up-regulated transcription factors in the brain versus blood in** ***S. pneumoniae*** **WCH16 and WCH43.**
(DOCX)

Table S4 **Comparison of gene expression between** ***S. pneumoniae*** **WCH16 and WCH43.**
(DOCX)

Table S5 ***In vitro*** **fitness of mutants versus wild-type** ***S. pneumoniae*** **WCH43 in a 1×1 competition.**
(DOC)

Table S6 **Primers for mutation and real-time RT-PCR validation.**
(DOCX)
