# Supplementary material for: A Transcription Factor Contributes to Pathogenesis and Virulence in Streptococcus pneumoniae
Source: PLoS One. 2013 Aug 13;8(8):e70862. doi: 10.1371/journal.pone.0070862 (PMC3742648; doi:10.1371/journal.pone.0070862)
Supplement: Table S1 — Highly up-regulated genes in S. pneumoniae WCH16 and WCH43 during pathogenesis. (DOCX) [file pone.0070862.s001.docx]

**Table S1** **Highly up-regulated genes in *S. pneumoniae* WCH16 and WCH43 during pathogenesis.**

| Gene **^a^** | Protein accession **^b^** | Protein Name | Lungs versus Nasopharnyx | Blood versus Lungs | Brain versus Blood |
| --- | --- | --- | --- | --- | --- |
| SP_0424 | P59201 | 3R-hydroxymyristoyl-ACP dehydratase (FabZ) | **√** |  | **√** |
| SP_0798 | P0A4H7 | DNA-binding response regulator (CiaR) | **√** |  | **√** |
| SP_0702 | P0CB78 | Orotate phosphoribosyltransferase (PyrE) | **√** |  | **√** |
| SP_1609 | Q97PK0 | Putative uncharacterized protein | **√** |  |  |
| SP_0779 | Q97RM4 | tRNA (guanine-N(1)-)-methyltransferase (TrmD) | **√** |  | **√** |
| SP_0788 | P67580 | Methionyl-tRNA synthetase (MetG) | **√** |  | **√** |
| SP_0780 | Q2MGI3 | ATP cone domain-containing protein | **√** |  | **√** |
| SP_0771 | Q97RN2 | cyclophilin type peptidyl-prolyl cis-trans isomerase | **√** |  | **√** |
| SP_0772 | Q97RN1 | Putative uncharacterized protein | **√** |  | **√** |
| SP_0773 | Q97RN0 | Putative uncharacterized protein | **√** |  | **√** |
| SP_0774 | Q97RM9 | Putative uncharacterized protein | **√** |  | **√** |
| SP_0781 | Q97RM3 | Putative uncharacterized protein | **√** |  | **√** |
| SP_0782 | Q97RM2 | Putative uncharacterized protein | **√** |  | **√** |
| SP_0787 | Q97RL7 | Putative uncharacterized protein | **√** |  | **√** |
| SP_0789 | Q97RL5 | Putative uncharacterized protein | **√** |  | **√** |
| SP_0790 | Q97RL4 | Putative uncharacterized protein | **√** |  | **√** |
| SP_0795 | Q97RK9 | Putative uncharacterized protein | **√** |  | **√** |
| SP_0796 | Q97RK8 | Putative uncharacterized protein | **√** |  | **√** |
| SP_0797 | Q97RK7 | Aminopeptidase | **√** |  | **√** |
| SP_0919 | Q97RA6 | Putative uncharacterized protein | **√** |  | **√** |
| SP_0927 | Q97R98 | LysR family transcriptional regulator | **√** |  | **√** |
| SP_0439 | Q97SE4 | Peptide chain release factor (PrfC) | **√** |  | **√** |
| SP_0429 | Q97SF3 | Putative uncharacterized protein | **√** |  | **√** |
| SP_0445 | Q97SD9 | Acetolactate synthase catalytic subunit | **√** |  | **√** |
| SP_0446 | Q97SD8 | Acetolactate synthase 3 regulatory subunit (IlvH) | **√** |  | **√** |
| SP_0675 | Q97RW1 | Short chain dehydrogenase/reductase family oxidoreductase | **√** |  | **√** |
| SP_0676 | Q97RW0 | Transcriptional regulator | **√** |  | **√** |
| SP_0677 | Q97RV9 | Putative uncharacterized protein | **√** |  | **√** |
| SP_0678 | Q97RV8 | Putative uncharacterized protein | **√** |  | **√** |
| SP_0683 | Q97RV3 | Putative uncharacterized protein | **√** |  | **√** |
| SP_0684 | Q97RV2 | Putative uncharacterized protein | **√** |  | **√** |
| SP_0685 | Q97RV1 | Putative uncharacterized protein | **√** |  | **√** |
| SP_0686 | Q97RV0 | Putative uncharacterized protein | **√** |  | **√** |
| SP_0691 | Q97RU6 | Putative uncharacterized protein | **√** |  | **√** |
| SP_0692 | Q97RU5 | Putative uncharacterized protein | **√** |  | **√** |
| SP_0693 | Q97RU4 | Putative uncharacterized protein | **√** |  | **√** |
| SP_0694 | Q97RU3 | Conserved domain protein | **√** |  | **√** |
| SP_0699 | Q97RT9 | Putative uncharacterized protein | **√** |  | **√** |
| SP_0423 | I6L8P7 | Acetyl-CoA carboxylase biotin carboxyl carrier protein subunit | **√** |  |  |
| SP_0424 | P59201 | 3R-hydroxymyristoyl-ACP dehydratase (FabZ) | **√** |  | **√** |
| SP_0967 | Q97R63 | Metalloprotease |  | **√** |  |
| SP_0569 | Q2MGH8 | Type II DNA modification methyltransferase |  | **√** |  |
| SP_2089 | Q2MGG5 | Transposase, IS1380-Spn1 related, truncation |  | **√** |  |
| SP_0800 | Q97RK6 | Putative uncharacterized protein |  | **√** |  |
| SP_1717 | Q97PC2 | ABC transporter, ATP-binding protein |  | **√** |  |
| SP_0325 | Q97SK7 | PTS system transporter subunit IID |  |  | **√** |
| SP_0326 | Q97SK6 | Preprotein translocase subunit YajC |  |  | **√** |
| SP_0327 | Q97SK5 | Putative uncharacterized protein |  |  | **√** |
| SP_0328 | Q97CV5 | IS1380-Spn1 transposase |  |  | **√** |
| SP_0313 | Q97SL7 | Glutathione peroxidase |  |  | **√** |
| SP_0333 | I6L8R8 | Transcriptional regulator |  |  | **√** |
| SP_0334 | P0CB58 | S-adenosyl-methyltransferase (MraW) |  |  | **√** |
| SP_0335 | I6L8V3 | Cell division protein FtsL |  |  | **√** |
| SP_0336 | P14677 | Penicillin-binding protein 2X |  |  | **√** |
| SP_0341 | Q97SJ8 | Putative uncharacterized protein |  |  | **√** |
| SP_0342 | Q54796 | Glucan 1,6-alpha-glucosidase |  |  | **√** |
| SP_0343 | Q97CV5 | IS1380-Spn1 transposase |  |  | **√** |
| SP_0344 | Q97SJ7 | IS630-Spn1, transposase Orf2 |  |  | **√** |
| SP_0349 | Q9AHD2 | Capsular polysaccharide biosynthesis protein Cps4D |  |  | **√** |
| SP_0350 | I6L8N6 | Capsular polysaccharide biosynthesis protein Cps4E |  |  | **√** |
| SP_0351 | Q97SJ5 | Capsular polysaccharide biosynthesis protein Cps4F |  |  | **√** |
| SP_0352 | I6L8X5 | Capsular polysaccharide biosynthesis protein Cps4G |  |  | **√** |
| SP_0421 | I6L8V8 | 3-ketoacyl-ACP reductase (FabG) |  |  | **√** |
| SP_0422 | I6L8U5 | 3-oxoacyl-ACP synthase |  |  | **√** |
| SP_0430 | Q97SF2 | Putative uncharacterized protein |  |  | **√** |
| SP_0431 | Q97SF1 | Putative uncharacterized protein |  |  | **√** |
| SP_0432 | IS1167 | Pseudogene |  |  | **√** |
| SP_0437 | Q97SE6 | Aspartyl/glutamyl-tRNA amidotransferase subunit A (GatA) |  |  | **√** |
| SP_0438 | Q97SE5 | Aspartyl/glutamyl-tRNA amidotransferase subunit C (GatC) |  |  | **√** |
| SP_0440 | Q6MML2 | Pseudogene |  |  | **√** |
| SP_0447 | Q97SD7 | Ketol-acid reductoisomerase |  |  | **√** |
| SP_0448 | Q97SD6 | Putative uncharacterized protein |  |  | **√** |
| SP_0579 | Q97S36 | Phenylalanyl-tRNA synthetase subunit alpha (PheS) |  |  | **√** |
| SP_0587 | Q97S29 | Putative uncharacterized protein |  |  | **√** |
| SP_0589 | Q97S27 | Serine acetyltransferase |  |  | **√** |
| SP_0590 | Q97S26 | Acetyltransferase |  |  | **√** |
| SP_0595 | Q97S21 | Putative uncharacterized protein |  |  | **√** |
| SP_0596 | Q97S20 | Putative uncharacterized protein |  |  | **√** |
| SP_0597 | Q97RP9 | Pseudogene |  |  | **√** |
| SP_0603 | I6L8W1 | DNA-binding response regulator VncR |  |  | **√** |
| SP_0605 | P0A4S1 | Fructose-bisphosphate aldolase |  |  | **√** |
| SP_0606 | Q97S17 | Oxidoreductase |  |  | **√** |
| SP_0676 | Q97RW0 | Transcriptional regulator |  |  | **√** |
| SP_0678 | Q97RV8 | Putative uncharacterized protein |  |  | **√** |
| SP_0683 | Q97RV3 | Putative uncharacterized protein |  |  | **√** |
| SP_0701 | P0CB75 | Orotidine 5'-phosphate decarboxylase |  |  | **√** |
| SP_0739 | I6L8N2 | MerR family transcriptional regulator |  |  | **√** |
| SP_0740 | Q97RQ8 | MutT/nudix family protein |  |  | **√** |
| SP_0741 | Q97RQ7 | Putative uncharacterized protein |  |  | **√** |
| SP_0742 | Q97RQ6 | Putative uncharacterized protein |  |  | **√** |
| SP_0747 | Q97RQ2 | Putative uncharacterized protein |  |  | **√** |
| SP_0748 | Q97RQ1 | Putative uncharacterized protein |  |  | **√** |
| SP_0749 | Q97RQ0 | Branched-chain amino acid ABC transporter substrate-binding protein |  |  | **√** |
| SP_0750 | Q97RP9 | Branched-chain amino acid ABC transporter permease |  |  | **√** |
| SP_0755 | Q2MGI2 | Peptide chain release factor 2 (PrfB) |  |  | **√** |
| SP_0756 | Q97RP4 | Cell division ABC transporter ATP-binding protein FtsE |  |  | **√** |
| SP_0757 | Q97RP3 | Cell division ABC transporter permease FtsX |  |  | **√** |
| SP_0758 | P35595 | PTS system transporter subunit IIABC |  |  | **√** |
| SP_0763 | Q97RN8 | Putative uncharacterized protein |  |  | **√** |
| SP_0764 | Q9X9S0 | Dihydroorotate dehydrogenase 1A |  |  | **√** |
| SP_0765 | Q97RN7 | DNA polymerase III subunit delta (HolA) |  |  | **√** |
| SP_0766 | P0A4J6 | Superoxide dismutase, manganese-dependent |  |  | **√** |
| SP_0784 | Q97RM0 | Glutathione reductase |  |  | **√** |
| SP_0797 | Q97RK7 | Aminopeptidase |  |  | **√** |
| SP_0904 | Q97RC0 | Putative uncharacterized protein |  |  | **√** |
| SP_0905 | Q97RB9 | Putative uncharacterized protein |  |  | **√** |
| SP_0906 | Q97RB8 | Putative uncharacterized protein |  |  | **√** |
| SP_0911 | Q97RB3 | Putative uncharacterized protein |  |  | **√** |
| SP_0912 | Q97RB2 | ABC transporter ATP-binding protein |  |  | **√** |
| SP_0913 | Q97RB1 | ABC transporter permease |  |  | **√** |
| SP_0919 | Q97RA6 | Putative uncharacterized protein |  |  | **√** |
| SP_0920 | Q97RA5 | Carboxynorspermidine decarboxylase |  |  | **√** |
| SP_0921 | Q97RA4 | Agmatine deiminase |  |  |  |

**^a^** Gene IDs were obtained from the *S. pneumoniae* TIGR4 (serotype 4) genome as deposited in the Kyoto Encyclopedia of Genes and Genomes (KEGG) database.

**^b^** Protein accession numbers were obtained from Universal Protein Resource (UniProt).
