# Supplementary material for: A Transcription Factor Contributes to Pathogenesis and Virulence in Streptococcus pneumoniae
Source: PLoS One. 2013 Aug 13;8(8):e70862. doi: 10.1371/journal.pone.0070862 (PMC3742648; doi:10.1371/journal.pone.0070862)
Supplement: Table S2 — List of top 50 significantly differentially-expressed genes as a result of SP_0927 mutation in S. pneumoniae WCH43 by microarray analysis. (DOCX) [file pone.0070862.s002.docx]

**Table S2** **List of top 50 significantly differentially-expressed genes as a result of SP_0927 mutation in *S. pneumoniae* WCH43 by microarray analysis.**

| **Up-regulated** | | **Down-regulated** | |
| --- | --- | --- | --- |
| **Gene ID** | **Average LOGratio** | **Gene ID** | **Average LOGratio** |
| SP_0927 | -3.956307449 | SP_0463 | 2.932951663 |
| SP_1817 | -2.335326041 | SP_2163 | 2.55904648 |
| SP_2003 | -2.220635328 | SP_0464 | 2.515616582 |
| SP_2005 | -2.042706732 | SP_2087 | 2.484663787 |
| SP_0617 | -1.999347882 | SP_2167 | 2.483314927 |
| SP_2002 | -1.901056749 | SP_0092 | 2.26789336 |
| SP_0798 | -1.89762411 | SP_1121 | 2.253585094 |
| SP_1027 | -1.887849401 | SP_1123 | 2.239057038 |
| SP_2072 | -1.876260095 | SP_2086 | 2.157366204 |
| SP_2001 | -1.793476996 | SP_0462 | 2.141156158 |
| SP_2004 | -1.77748376 | SP_0061 | 2.112220556 |
| SP_0501 | -1.773983932 | SP_1685 | 2.083286129 |
| SP_1898 | -1.771403826 | SP_2166 | 1.996267163 |
| SP_2009 | -1.760828254 | SP_2143 | 1.988948711 |
| SP_2181 | -1.719331103 | SP_1684 | 1.964807553 |
| SP_1714 | -1.694599596 | SP_1122 | 1.899247286 |
| SP_2008 | -1.690549429 | SP_2088 | 1.88194258 |
| SP_2238 | -1.68547697 | SP_1580 | 1.842944662 |
| SP_1897 | -1.681563064 | SP_0062 | 1.766272296 |
| SP_1012 | -1.665714761 | SP_1683 | 1.751248595 |
| SP_0584 | -1.655129429 | SP_2084 | 1.718728235 |
| SP_0102 | -1.653809553 | SP_1199 | 1.673609317 |
| SP_1992 | -1.635446135 | SP_2162 | 1.671044662 |
| SP_0974 | -1.623867953 | SP_2148 | 1.669784711 |
| SP_1010 | -1.588834141 | SP_2085 | 1.657116576 |
| SP_0861 | -1.585897118 | SP_0703 | 1.635487284 |
| SP_0799 | -1.568172331 | SP_0461 | 1.594777458 |
| SP_0258 | -1.568134013 | SP_2055 | 1.529993377 |
| SP_0531 | -1.560334968 | SP_0715 | 1.516719775 |
| SP_2239 | -1.52643276 | SP_2109 | 1.505643274 |
| SP_0023 | -1.521667568 | SP_0712 | 1.465141483 |
| SP_0677 | -1.521007118 | SP_0063 | 1.451059045 |
| SP_2007 | -1.5169152 | SP_1197 | 1.443073377 |
| SP_0486 | -1.514078581 | SP_0060 | 1.416549848 |
| SP_0082 | -1.505997633 | SP_1124 | 1.39527936 |
| SP_1271 | -1.46054446 | SP_0064 | 1.379916082 |
| SP_0346 | -1.451981198 | SP_0646 | 1.356874536 |
| SP_1894 | -1.437465855 | SP_0268 | 1.352354264 |
| SP_1353 | -1.435157644 | SP_0065 | 1.34868947 |
| SP_0959 | -1.427566521 | SP_0285 | 1.329881617 |
| SP_0742 | -1.427454735 | SP_0320 | 1.313525083 |
| SP_0022 | -1.416147316 | SP_1693 | 1.264838441 |
| SP_1788 | -1.413995063 | SP_1682 | 1.257745611 |
| SP_2067 | -1.410106085 | SP_0645 | 1.251911908 |
| SP_1625 | -1.409202742 | SP_0067 | 1.220163244 |
| SP_0564 | -1.388861041 | SP_0091 | 1.212177814 |
| SP_0021 | -1.387275828 | SP_0068 | 1.165941246 |
| SP_2107 | -1.380761274 | SP_2182 | 1.162454252 |
| SP_0503 | -1.378433539 | SP_2110 | 1.127369397 |
| SP_2225 | -1.372900757 | SP_0066 | 1.115608417 |
