# Supplementary material for: A Transcription Factor Contributes to Pathogenesis and Virulence in Streptococcus pneumoniae
Source: PLoS One. 2013 Aug 13;8(8):e70862. doi: 10.1371/journal.pone.0070862 (PMC3742648; doi:10.1371/journal.pone.0070862)
Supplement: Table S3 — List of up-regulated transcription factors in the brain versus blood in S. pneumoniae WCH16 and WCH43. (DOCX) [file pone.0070862.s003.docx]

**Table S3 List of up-regulated transcription factors in the brain versus blood in *S. pneumoniae* WCH16 and WCH43.**

| Gene**^A^** | Protein accession**^B^** | Protein name |
| --- | --- | --- |
| SP_2077 | P0A2Y2 | Arginine repressor (ArgR) |
| SP_1584 | Q97PM1 | transcriptional repressor (CodY) |
| SP_1219 | P72524 | DNA gyrase subunit A (GyrA) |
| SP_0515 | Q9X4R2 | Heat-inducible transcription repressor (HrcA) |
| SP_2112 | P0A4T1 | Maltose operon transcriptional repressor (MalR) |
| SP_0855 | P72525 | DNA topoisomerase IV subunit A (ParC) |
| SP_1073 | P0A4I9 | RNA polymerase sigma factor (RpoD) |
| SP_0014 | Q97CV2 | Transcriptional regulator ComX1 |
| SP_2006 | Q8CM18 | Transcriptional regulator ComX2 |
| SP_0739 | I6L8N2 | MerR family transcriptional regulator |
| SP_2000 | I6L8S5 | DNA-binding response regulator (TCS11) |
| SP_2172 | I6L8N3 | Adc operon repressor (AdcR) |
| SP_0661 | I6L8X3 | DNA-binding response regulator (TCS09) |
| SP_1854 | Q97NZ5 | Galactose operon repressor (GalR) |
| SP_0333 | I6L8R8 | Transcriptional regulator (YorfE) |
| SP_0395 | Q97SH3 | Transcriptional regulator |
| SP_0058 | Q97T92 | GntR family transcriptional regulator |
| SP_0926 | Q97R99 | Putative uncharacterized protein |
| SP_0676 | Q97RW0 | Transcriptional regulator |
| SP_1856 | Q97NZ3 | MerR family transcriptional regulator |
| SP_2168 | Q97N87 | Fucose operon repressor |
| SP_2062 | Q97NG7 | MarR family transcriptional regulator |
| SP_1203 | Q97QK2 | Transcriptional repressor (ArgR2) |
| SP_0893 | Q97RC9 | Transcriptional repressor (ArgR1) |
| SP_0330 | Q97SK3 | Sugar binding transcriptional regulator (RegR) |
| SP_1331 | Q97Q94 | Phosphosugar-binding transcriptional regulator (RpiR) |
| SP_0875 | Q97RE7 | Lactose phosphotransferase system repressor (LacR1) |
| SP_0416 | Q97SF5 | MarR family transcriptional regulator |
| SP_1393 | Q97Q38 | Putative uncharacterized protein |
| SP_1446 | Q97PZ2 | GntR family transcriptional regulator |
| SP_0927 | Q97R98 | LysR family transcriptional regulator |
| SP_0006 | Q97TD0 | Transcription-repair coupling factor (Mfd) |
| SP_1920 | Q97NU2 | MarR family transcriptional regulator |
| SP_2020 | Q97NK6 | GntR family transcriptional regulator |
| SP_0246 | Q97SS8 | DeoR family transcriptional regulator |

**^a^** Gene IDs were obtained from the *S. pneumoniae* TIGR4 (serotype 4) genome as deposited in the Kyoto Encyclopedia of Genes and Genomes (KEGG) database.

**^b^** Protein accession numbers were obtained from Universal Protein Resource (UniProt).
