# Supplementary material for: A Transcription Factor Contributes to Pathogenesis and Virulence in Streptococcus pneumoniae
Source: PLoS One. 2013 Aug 13;8(8):e70862. doi: 10.1371/journal.pone.0070862 (PMC3742648; doi:10.1371/journal.pone.0070862)
Supplement: Table S4 — Comparison of gene expression between S. pneumoniae WCH16 and WCH43. (DOCX) [file pone.0070862.s004.docx]

**Table S4 Comparison of gene expression between *S. pneumoniae* WCH16 and WCH43.**

| **Strain** |  | **Number of up-regulated genes** | | |
| --- | --- | --- | --- | --- |
| WCH43 | Lungs vs Nasopharynx |  | 31 |  |
|  | Blood vs Lungs |  | 16 |  |
|  | Brain vs Blood |  | 117 |  |
| WCH16 | Lung vs Nasopharynx |  | 84 |  |
|  | Blood vs Lungs |  | 3 |  |
|  | Brain vs Blood |  | 108 |  |
