# Supplementary material for: A Transcription Factor Contributes to Pathogenesis and Virulence in Streptococcus pneumoniae
Source: PLoS One. 2013 Aug 13;8(8):e70862. doi: 10.1371/journal.pone.0070862 (PMC3742648; doi:10.1371/journal.pone.0070862)
Supplement: Table S5 — In vitro fitness of mutants versus wild-type S. pneumoniae WCH43 in a 1×1 competition. (DOC) [file pone.0070862.s005.doc]

| **Strain** | **fitness ± s.e.m.** | |
| --- | --- | --- |
|  | **1.5 h** | **3 h** |
| Wild-type | 1 | 1 |
| ΔSP_0746 | 1.056±0.094 | 1.050±0.070 |
| ΔSP_0927 | 1.277±0.006 | 0.975±0.095 |

**Table S5** ***In vitro* fitness of mutants versus wild-type *S. pneumoniae* WCH43 in a 1 × 1 competition.**
