# Supplementary material for: A Transcription Factor Contributes to Pathogenesis and Virulence in Streptococcus pneumoniae
Source: PLoS One. 2013 Aug 13;8(8):e70862. doi: 10.1371/journal.pone.0070862 (PMC3742648; doi:10.1371/journal.pone.0070862)
Supplement: Table S6 — Primers for mutation and real-time RT-PCR validation. (DOCX) [file pone.0070862.s006.docx]

**Table S6.** **Primers for mutation and real-time RT-PCR validation.**

**Primers for mutation**

| **Primer Name** | **Sequence 5' to 3'** |
| --- | --- |
| SP_0746 Flank F | CATTCCTTCTGGAGCTCAGCCATCG |
| SP_0746 Flank R | GCTTGTTTGACCAGTTACACCTTC |
| SP_0746 Ery X | TTGTTCATGTAATCACTCCTTCGAGTTTTAATTTTGTTGGTCAAATGAC |
| SP_0746 Ery Y | CGGGAGGAAATAATTCTATGAGAAGGCAAACTCGACTGGGTTTGC |
| SP_0746 Upseq | TCTCAGAAAATCAAACCAAGAATCC |
| SP_0927 Flank F | AGGAGGGTGCCGTCCATATCCAAG |
| SP_0927 Flank R | CAAGAATAATCGCATCACTGCCACCTTC |
| SP_0927 Ery X | TTGTTCATGTAATCACTCCTTCTTCTTTATCTTTTCATTATACCATATTTTA |
| SP_0927 Ery Y | CGGGAGGAAATAATTCTATGAGCAATAGTGGCAGTCATTGTACTGC |
| SP_0927 Upseq | CTCCACTGAATCGTTGGCAAAGC |
|  |  |
| **Primers for real-time RT-PCR validation** | |
| **Primer Name** | **Sequence 5' to 3'** |
| SP_0246 F | ATCTGGATCGACCTGTGCTT |
| SP_0246 R | CCCAACAAAAGCTTGACACA |
| SP_0313 F | TCAGGGCTTCGAAATATTGG |
| SP_0313 R | TTCCATTCGACTCGTTTTCC |
| SP_0368 F | TCGTGAAGCTGGTGATCAAG |
| SP_0368 R | TCGACACCACCCACTACAAA |
| SP_0463 F | ATGCGCATGTGTATCCAAAA |
| SP_0463 R | TCACCTGCTTCAAGTGCAAC |
| SP_0486 F | GCTGGATTGGATTACTGGGA |
| SP_0486 R | TTCATGGGAATACGGAGAGC |
| SP_0515 F | AAAAGGTGGCCTTGGAGATT |
| SP_0515 R | CGGTTGACCACATTGACTTG |
| SP_0584 F | GCGAGTTGATTGAGGCTAGG |
| SP_0584 R | CCTGTTCAAGTGGGACGATT |
| SP_0676 F | TCTTTATCCGCAATCCCAAG |
| SP_0676 R | AAAGGCATTGACCACAAAGG |
| SP_0746 F | GCTTTTCTTGGATGCCCAA |
| SP_0746 R | CCCATAGATGCAGCCATTCC |
| SP_0766 F | GCATTTGCACCATGACAAAC |
| SP_0766 R | AGGCTGCTTGGAATTCTTCA |
| SP_0784 F | GGCTTGCAACTTGAAAAAGC |
| SP_0784 R | AGTTCCGATAGCAGGGTGTG |
| SP_0798 F | GATGGAGAAGAAGGTC |
| SP_0798 R | GTCATAATCAGAACTGG |
| SP_0927 F | TATCTCCGTGAGGGTCATCC |
| SP_0927 R | TCCGCTCCAAAATACCATTC |
| SP_1123 F | ATTGCGCTATGTTTTACGGG |
| SP_1123 R | TGGCTCTTCGTTTTTGACCT |
| SP_1609 F | CCATTGAAAAGGGTGTGGAC |
| SP_1609 R | CAATTCCACGTTCTGGACCT |
| SP_1683 F | TACAGGTGCTTTCCCAGTCC |
| SP_1683 R | GCTGGTTTTTCGTCACCATT |
| SP_1717 F | ACGGACCAGTTCAGGATGTC |
| SP_1717 R | GATAGTTGGCGCTTGTTGGT |
| SP_1856 F | GTTGGGAATTTCAGCGGATACGAT |
| SP_1856 R | TCCTTCCTTATAAAGTTTAATTTTGAG |
| SP_2000 F | AGTATTCGCCTGAATTGATGG |
| SP_2000 R | ACATAGTTTCGGACTGTTCCG |
| SP_2006 F | TTCATTTATGGGAATTGTCGG |
| SP_2006 R | GTATCTACGCTTCTGACTTTCC |
| SP_2072 F | CGTCGAGAGCGATGATTACA |
| SP_2072 R | CATTGACAAGTTGGACACCG |
| SP_2084 F | CGCTGTTAAGGAGAAAACCG |
| SP_2084 R | CGTCTGCAAGTTCAGCCATA |
| SP_2163 F | GATTTCTGCACCGGTAGGTT |
| SP_2163 R | CACCTCCCGTTACCTTTTCA |
| SP_2172 F | GCGGAAAATCAGCATGAAAT |
| SP_2172 R | TTTCCAACATCCCTTCCTTG |
